# The effect of BKV reactivation on cytokines behavior in kidney transplanted patients

**DOI:** 10.1186/s12882-021-02645-y

**Published:** 2022-01-07

**Authors:** Zahra Rahimi, Ramin Yaghobi, Afsoon Afshari, Jamshid Roozbeh, Mohammad Javad Mokhtari, Ali Malek Hosseini

**Affiliations:** 1 Department of Biology, Zarghan branch, Islamic Azad University, Zarghan, Iran; 2 Shiraz Transplant Research Center, Shiraz University of Medical Sciences, Shiraz, Iran; 3 Shiraz Nephro-Urology Research Center, Shiraz University of Medical Sciences, Shiraz, Iran

**Keywords:** IL-27, Polyomavirus BK, Kidney, Transplantation

## Abstract

**Background:**

BK virus associated nephropathy (BKVAN) is one of the common causes of graft loss among kidney transplanted recipients (KTRs). The current treatment for BKV nephropathy is decreasing the immunosuppressive regimen in KTRs. Interleukin-27 (IL-27) is a multifunctional cytokine that might be the front-runner of an important pathway in this regard. Therefore, in current study it is tried to evaluate the changes in the expression level of IL-27 and some related molecules, resulting from BKV reactivation in KTR patients.

**Methods:**

EDTA-treated blood samples were collected from all participants. Patients were divided into two groups, 31 kidney transplant recipients with active and 32 inactive BKV infection, after being monitored by Real time PCR (Taq-Man) in plasma. Total of 30 normal individuals were considered as healthy control group. Real time PCR (SYBR Green) technique is used to determine the expression level of studied genes.

**Results:**

The results of gene expression comparisons showed that the expression level of IL-27, IFN-γ, TNF-α, TNFR2 and IRF7 genes was significantly higher in inactive group in comparison to active group. The expression level of TLR4 was lower in both active and inactive groups in comparison to control group. ROC curve analysis showed that IL-27 and IRF7 are significantly different amongst other studied genes. Finally, the analyses revealed that the expression level of most of the studied genes (except for TNF-α and TLR4) have significant correlation with viral load.

**Conclusions:**

Our findings revealed that IL-27, IFN-γ, TNF-α, TNFR2 and IRF7 expression level is higher in inactive group and TLR4 expression level is lower in patients’ groups in comparison to control group. Also, ROC curve analysis showed IL-27 and IRF7 can significantly differentiate studied groups (BKV active vs. inactive). Therefore, these results might help elucidating the pattern in charge of BKV reactivation in kidney transplanted patients.

**Supplementary Information:**

The online version contains supplementary material available at 10.1186/s12882-021-02645-y.

## Introduction

Due to immunosuppression regimens followed by kidney transplantation (KT), clinically significant reactivation of latent BK polyomavirus (BKV) occurs (median: 19.5% of KT recipients) post-transplantation that might be followed by development of BKV-associated nephropathy (BKVAN). BKVAN is accompanied with a significant risk of allograft loss [1–3]. It is documented that BKV reactivation happens in some kidney transplant recipients (KTR) but some of them do not experience this reactivation; although all of the patients have the same immunosuppressive regimen [4, 5]. The first barrier for BKV infection is innate immunity which does not seem prevalent enough to clear viral infection. For instance, Natural killer (NK) cells and Dendritic cells (DCs) have important roles against viral infections [6, 7] through coordinating the adaptive immune responses that facilitate the direct killing of infected cells [8]. By the way, activating the antigen presenting adaptive immune responses failed to clear or control the BKV infection [9, 10].

By considering the mentioned points about innate immunity in clearance of BKV, especially in KTRs, the role of acquired immunity becomes more noticeable. Commonly in viral infections such as BKV, BKV-specific antibody responses might play an important role in neutralizing circulating viruses, but antibodies alone are not able to control latent persistent infection [11]. The effective control of latent viral reactivation might appear to be dependent to the induction of constant antiviral memory T cell responses [12]. Soon after primary exposure to antigens of BK virus, humoral response of adaptive immunity through production of neutralizing antibodies starts to control the virus infection. However, mutations in viral receptors offers an escape from immune system [13, 14]. Studies show that polyfunctional specific BKV T cell (both CD4 and CD8) responses are important for BKV viruria and viremia in KTRs [15–21]. Still, it seems that CD4 T cells are more potent in response to BKV-infected KTRs [22–24]. Also, it is detected that CD4 T cells might have direct controlling capacity through the expression of pro-inflammatory cytokines, including gamma interferon (IFN-γ) and tumor necrosis factor (TNF) and granzyme B molecules. These activities can be executed when there is a lack of CD8 T cell immunity [25].

Cytokines are important mediators between innate and adaptive immunity. A number of factors may be considered in this regard, one of which is immune system capacity and pro- and anti-inflammatory cytokines function. In the immune system, one of the mysterious cytokines is interleukin-27 (IL-27). IL-27 is a heterodimeric cytokine that is composed of IL-27p28 and Epstein-Barr virus induced gene 3 (EBI3) [4, 5]. Although some scientific literatures have demonstrated pro-inflammatory role for IL-27 that cause development in TH1 cell responses, there are some newer evidences that clarify the antagonizing effects of this cytokine over T cell responses [26]. IL-27 induces its effect through attachment to its receptor (IL-27R; WSX-1), its expression is detected on a wide range of cells (macrophages, NK, B, CD4 and CD8 T cells) and also during development of CD4 T cells into both Th1 and Th2 cells. This means that IL-27 is necessary for early CD4 T cell differentiation [27]. Studies indicate that the initial production of IFN-γ following infection is maintained through IL-27 [28].

There are small studies on the importance of the pre- and post-activating pathway of IL-27 in viral infections. Previously, the anti-inflammatory role of IL-27, through effective production of IFN-γ was studied for prevention of viral infections such as HBV [29], HCV and HCV/HIV co-infection [30, 31]. There is no study on the IL-27 pathway in BK infected KT patients. Considering the importance of pro- and anti-inflammatory effects of cytokines, in this research, the behavior of IL-27 from the point of gene expression is studied. Therefore, to have a more extended view, the up-stream molecules such as toll-like receptor (TLR) 3 and 4, interferon regulatory transcription factor (IRF) 3 and 7 and down-stream molecules such as IL-27R, IFN-γ, tumor necrosis factor- alpha (TNF-α) and its receptors: TNF-αRA and TNF-αRB, in IL-27 stimulating pathway are studied.

## Materials and methods

### Patients

The current study was conducted on selected 63 kidney transplant recipients who were admitted to the Transplant Ward, Namazi Hospital, (affiliated to Shiraz University of Medical Sciences, Shiraz, Iran) between 2016 and 2019 (mean of kidney transplantation per year = 350). EDTA-treated blood samples were collected from each patient. Patients were divided into two groups, 31 kidney transplant recipients with active (named active group) and 32 inactive BKV infection (named inactive group).

All patients’ samples were monitored for detecting the presence of BKV infection in plasma by Taq-Man Real time PCR protocol. BKV active samples were selected from the kidney transplanted patients who were admitted to the hospital during 6 months to 24 months (mean = 15 months) after transplantation for unreasonable creatinine rise. Biopsy and SV40 staining is done for patients who are suspected to BK nephropathy. The blood samples were taken from them and after confirmation of BKV infection through real-time PCR and biopsy, the selected samples included in the study. BKV viremia is accepted as a standard index for PVN diagnosis having diagnostic cut-off values that vary between 10^3^ and 10^4^ copies/mL of BKV genomic DNA for PVAN in plasma samples (the viral load and other related data is presented in Table S1). All the samples taken from KTRs with BKV reactivation were collected before starting the treatment strategies. Furthermore, all the samples were followed for signs of rejection and the samples with biopsy proven rejection signs at the time of sampling were excluded from the study. Also, studied patients was selected among the patients who received the first transplantation and collected patients’ samples were non-sensitized (Luminex flow PRA negative).

All patients were given the same routine regimen of immunosuppressive drugs which consisted of tacrolimus or cyclosporine with mycophenolate mofetil and steroids. The blood level of 200 mg/mL was considered the therapeutic target for CsA (5 mg/kg/d) or 10 mg/mL for tacrolimus. The buffy coat and plasma of all samples were separated using Ficol (Nycomed, Zurich, Switzerland) gradient for further analysis.

**Fig. 1:**
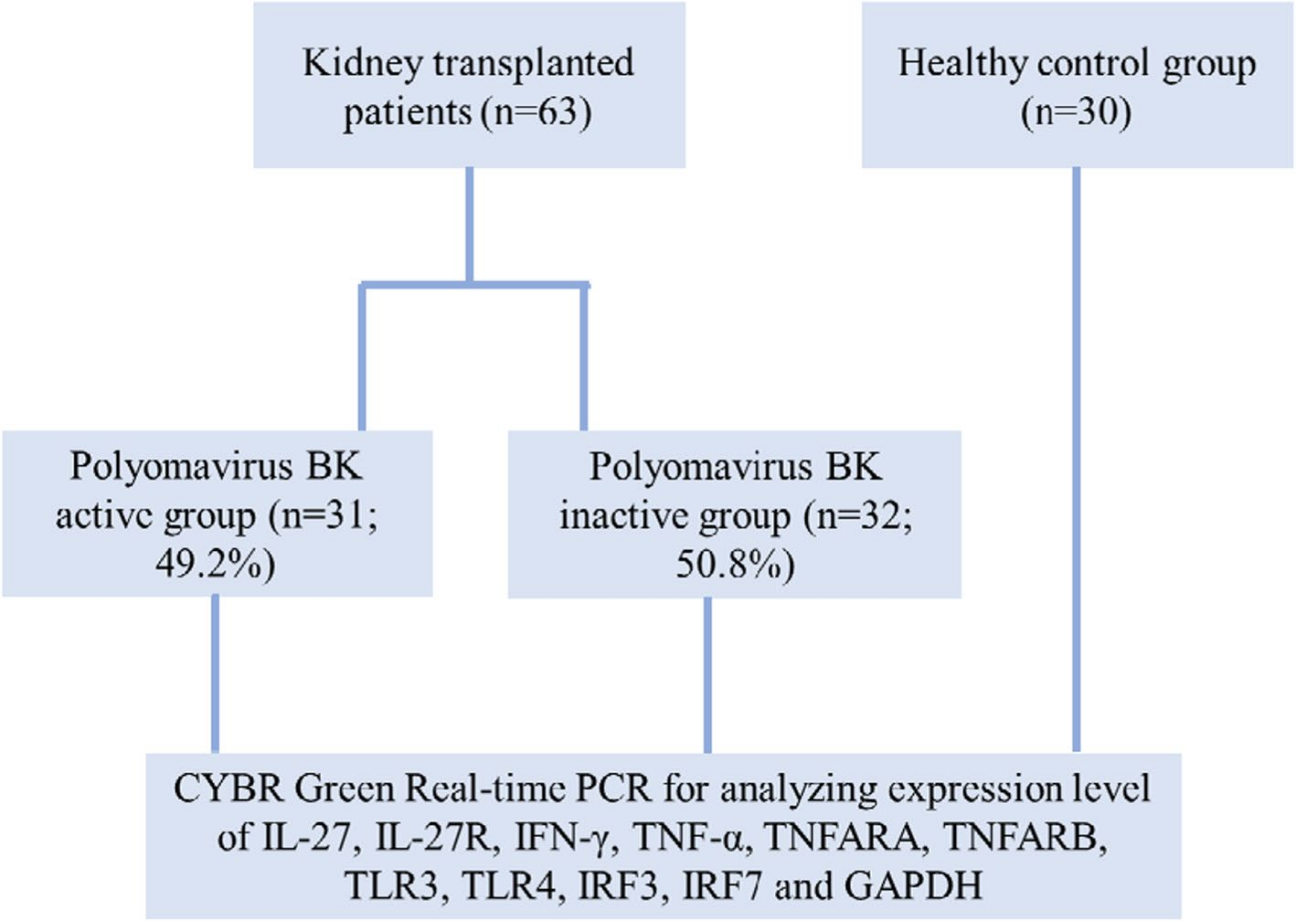
Flow chart of patient numbers and information

Also, 30 normal individuals without any active infection or other inflammatory diseases were considered as healthy control group. This study was approved by the Ethics Committee of Shiraz University of Medical Sciences. The protocols used were in conformity with the ethical guidelines of Declaration of Helsinki. Informed consent was obtained from all participant patients. Donors were selected from cadavers and were chosen based on compatibility of blood ABO group. Information about the number of patients is shown in the flow chart (Fig. 1).

### Virology

#### Polyomavirus BK detection

In order to screen the patients for BKV infection, Taq-Man Real-time PCR (ABI, Step One Plus, USA) assay was done. Invisorb Spin Virus DNA Blood Mini-kit (Invitek, Germany) was used for DNA extraction from all collected samples according to the manufacturer’s instruction. 200 μl of each plasma sample was separated for DNA extraction. The concentration and purity of extracted DNA samples were estimated by measuring the optimal density at 260 and 280 nm, using a NanoDrop spectrophotometer (Thermofisher Scientific, USA). The BKV load was determined for all samples by Real-time PCR instrument using BKV Taq-Man Real Time PCR kit (Gensig, Primer Design, England) according to the manufacturer’s protocols, respectively.

All the used samples were negative for cytomegalovirus (CMV), hepatitis B (HBV) and C (HCV) and human immunodeficiency virus (HIV).

### Cytokine gene expression analysis

#### RNA extraction and cDNA synthesis

RNX plus solution (Cinna Gen, Iran) was used for total RNA extraction from each sample buffy coat according to the manufacturer’s instruction. Also, optimal density in 260/280 nm was evaluated for each extracted RNA sample to determine the concentration and purity of them. Finally, RNA integrity was evaluated by electrophoresis on 1% agarose gel. After RNA extraction, the cDNA for each sample was synthesized using Takara cDNA synthesis kit (Takara, Dalian, Japan). According to the manufacturer’s instructions, 500 ng of RNA extracted from each sample was used for this reaction.

#### SYBR Green Real-time PCR

For measuring the RNA expression level, SYBR Green Real-time PCR was performed using SYBR Premix Ex TaqII kit (Takara, Dalian, Japan). In order to prevent amplification of genomic DNA contaminations, the forward and reverse primers were designed in exon junctions and their sequences are shown in Table 1. Also, for normalizing the results of each target gene, Glyceraldehyde-3-Phosphate Dehydrogenase (GAPDH) gene expression level was evaluated in each sample. The reaction mixture was prepared according to manufacturer’s protocol. Briefly, 10 μl of SYBR Green Premix, 0.8 μl of 10 pm of each forward and reverse primer, 0.4 μl of SYBR Green Dye, 2 μl of each DNA template were added and the reaction reached 20 μl by adding DEPC-water. The program for amplification of each primer pair and the primer sequences are shown in Table 1. All the runs were followed by melting-curve analysis in order to verify the specificity of the amplification reaction.

**Table 1:** The sequences of the forward and reverse primers and PCR condition

Gene name	mRNA ID	Primers	Length (bp)	Annealing temperature (°C)	PCR condition
**IRF3**	NM_001197122.2	Forward: 5′- TTGGGGACTTTTCCCAGCC Reverse: 5′- TCCAGAATGTCTTCCTGGGT	82	58	**1 cycle:** 95 °C /5 min., **40 cycles:** 95 °C/30 s 20 s/ annealing 72 °C/30 s Followed by: melt curve
**IRF7**	NM_001572.5	Forward: 5′- GTGAGGGTGTGTCTTCCCTG Reverse: 5′- TCGTCATAGAGGCTGTTGGC	73		
**GAPDH**	NM_001357943.2	Forward: 5′-GGACTCATGACCACAGTCC Reverse: 5′-CCAGTAGAGGCAGGGATGAT	119		
**IFN-γ**	NM_000619.3	Forward: 5′- CAGCTCTGCATCGTTTTGGG Reverse: 5′- TCCGCTACATCTGAATGACCTG	110		
**TLR3**	NM_003265.3	Forward: 5′-GGGCAAGAACTCACAGGCCAGG Reverse: 5′-AAGGGCCACCCTTCGGAGCA	147		
**IL-27R**	NM_004843.4	Forward: 5′- CGGAGCTGAAGACCATACCC Reverse: 5′- CGCCCGACAAATCCTCTTCT	114	59	
**TNFARB**	NM_001066.3	Forward: 5′- CACATGCCGGCTCAGAGAAT Reverse: 5′- AGCTGGGTGTATGTGCTGTC	144		
**TLR4**	NM_003266.4	Forward: 5′- TCAAGCCAGGATGAGGACTGGGT Reverse: 5′- CAGCAATGGCCACACCGGGA	118		
**IL-27**	NM_145659.3	Forward: 5′- GTGAACCTGTACCTCCTGCC Reverse: 5′- CGTGGTGGAGATGAAGCAGA	111	60	
**TNFARA**	NM_001065.4	Forward: 5′- GAGAGGCCATAGCTGTCTGG Reverse: 5′-CTCTCACACTCCCTGCAGTC	124		
**TNF-α**	NM_001065.4	Forward: 5′-CTTCTGCCTGCTGCACTTTG Reverse: 5′- CTACAGGCTTGTCACTCGGG	128	61	

### 2–4- statistical analysis

All data were collected in EPSPS ver. 22 (SPSS, Chicago, IL, USA). The mRNA expression level of each studied gene was calculated using Livak (2-*ΔΔCt*) method. The nonparametric tests were performed for analyzing the difference of expression levels between different groups of patients. Also, Spearman correlation analysis (two-sided) was used to evaluate the relation between variables (GraphPad Software, Prism 6.01, CA, USA). ROC curve analysis, sensitivity, and specificity of studied genes were determined using MedCalc Statistical Software version 17.9 (MedCalc Software, Ostend, Belgium). Finally, *p* < 0.05 was considered as statistically significant.

## Results

### Demographic details of patients

The age range of 63 kidney transplanted patients participating in this study was 18 to 68 years (mean ± SD: 43 ± 7.8 years). The patients were divided into two groups: 31 kidney transplant recipients with active infection with the age range of 21 to 63 years (mean ± SD: 44.16 ± 8.32 years) and 32 without active BKV infection with the age range of 18 to 68 years (mean ± SD: 40. 41 ± 9.9 years). The normal group was composed of 30 persons with the age between 28 and 60 and was composed of 15 male and 15 females.

### Comparing studied gene expression in BKV inactive and active kidney transplant recipients

**Fig. 2:**
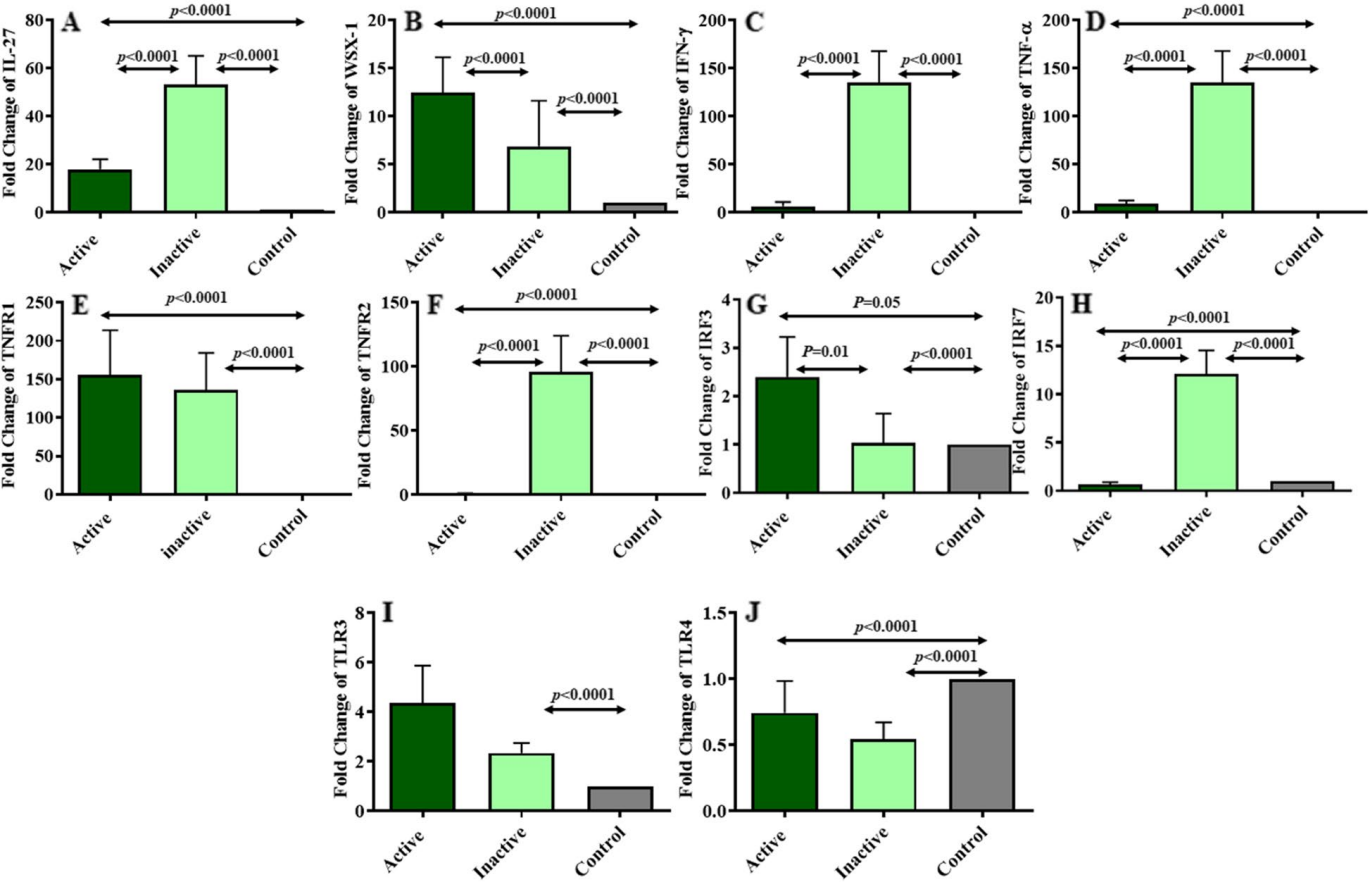
The expression level of studied genes compared in the active, inactive, and control groups

The expression level of the studied genes was compared in the three studied groups (active, inactive, and control). This analysis is shown in Fig. 2. The statistical analyses showed that the mean expression level of studied genes is relatively significant between inactive and control groups. The results of gene expression comparisons showed that the expression level of IL-27, IFN-γ, TNF-α, TNFR2 and IRF7 genes (Fig. 2A, C, D, F and H) was significantly higher in inactive group in comparison to active group. The expression pattern of other studied genes such as WSX-1, TNFRI, IRF3 and TLR3 (Fig. 2B, E, G and I) was higher in active group comparing to inactive one; In WSX-1 and IRF3 this increase was statistically significant. Finally, the expression level of TLR4 (Fig. 2J) was lower in both active and inactive groups in comparison to control group.

### The receiver operating characteristic (ROC) curve analysis of expression level of studied genes between active and inactive groups of patients

**Fig. 3:**
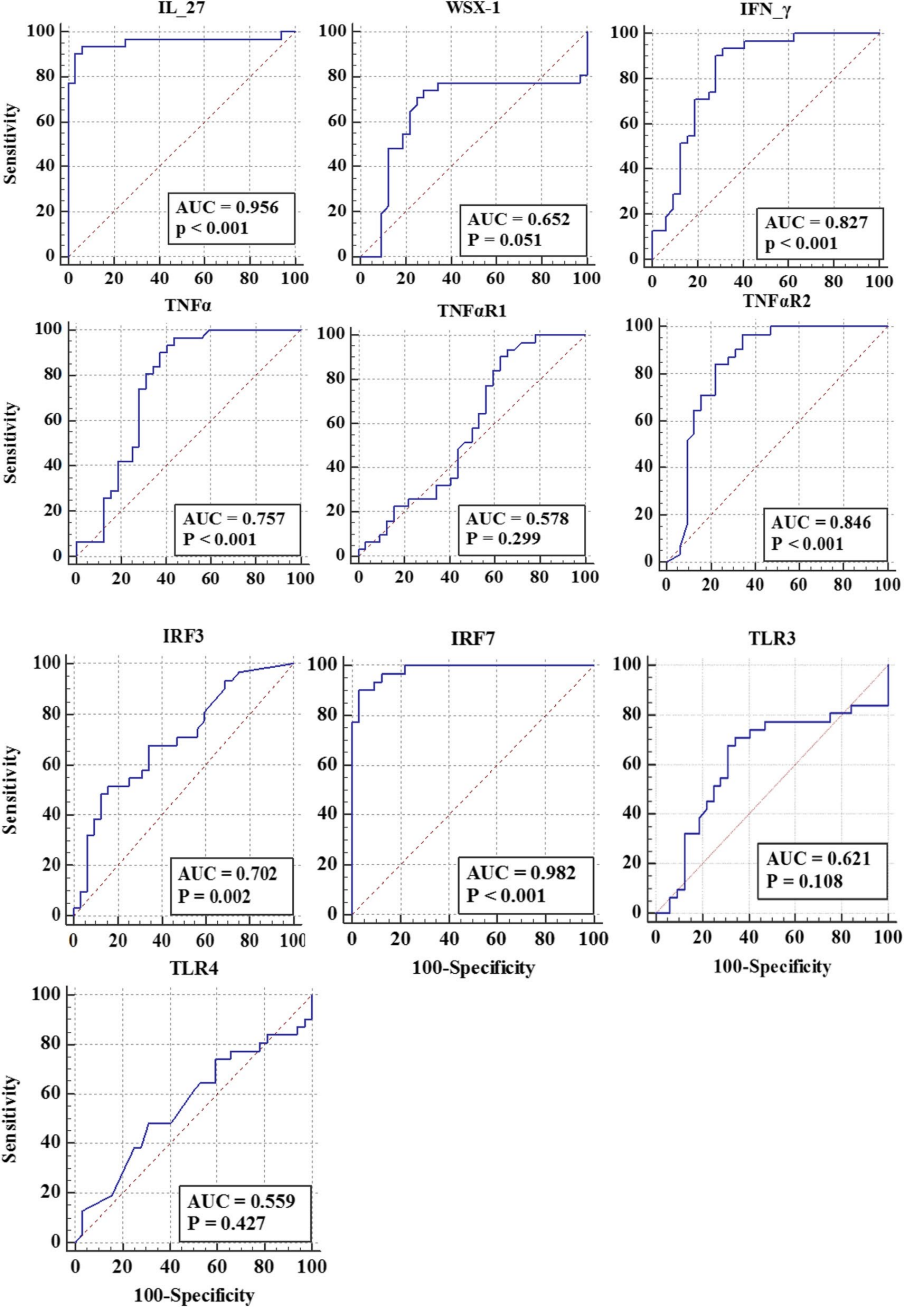
The ROC curve of studied genes

The ROC curve for showing the performance of a classification model at all classification thresholds and AUC (Area Under the ROC Curve) for measuring the entire two-dimensional area underneath the entire ROC curve analysis of studied genes are shown in Fig. 3 and Table 2, respectively. These results showed that IL-27 (*p* < 0.001 and AUC = 0.956) and IRF7 (*p* < 0.001 and AUC = 0.982) might have more important roles amongst other suited genes.

**Table 2:** The AUC, *p* value, cut-off value, sensitivity and specificity of studied genes

Gene Name	AUC (95% CI)	***p*** Value	Cut Off Value	Sensitivity (95% CI)	Specificity (95% CI)
**IL-27**	0.956 (0.872–0.991)	≤0.001	≤6.5	93.55 (78.6–99.2)	93.75 (79.2–99.2)
**IL-27R (WSX-1)**	0.652 (0.522–0.868)	0.051	≤3.84	74.19 (55.4–88.1)	71.87 (53.3–86.3)
**IFN-γ**	0.827 (0.711–0.911)	≤0.001	≤9.65	93.55 (78.6–99.2)	68.75 (50–83.9)
**TNF-α**	0.757 (0.632–0.856)	≤0.0001	≤36.76	96.5 (78.6–99.2)	68.75 (50–83.9)
**TNFR1**	0.578 (0.447–0.701)	0.299	> 3.92	93.55 (78.6–99.2)	34.38 (18.6–53.2)
**TNFR2**	0.846 (0.733–0.924)	≤0.0001	≤3.46	96.77 (83.3–99.9)	65.62 (46.8–81.4)
**IRF3**	0.702 (0.574–0.811)	0.002	> 0.44	51.61(33.1–69.8)	84.37 (67.2–94.7)
**IRF7**	0.982 (0.911–0.999)	≤0.0001	≤2.13	90.32 (74.2–98.0)	96.87 (83.8–99.9)
**TLR3**	0.621 (0.490–0.741)	0.108	≤1.36	70.97 (52.0–85.8)	65.62 (46.8–81.4)
**TLR4**	0.559 (0.428–0.684)	0.427	≤0.14	48.39 (30.2–66.9)	68.75 (50.0–83.9)

### Comparing lab indexes between blood factors and studied genes in active and inactive patient groups

The results of studying the relation between blood factors with different gene expression in both groups of active and inactive groups were done using Spearman method. The results showed that the expression pattern of IFN-γ in active patient group showed a significant relationship (*p* = 0.02) with BUN concentration. Also, in inactive group a significant relationship (*p* = 0.04) was detected with Serum Na. 

### Study of relationships between viral load and studied genes in active group

**Fig. 4:**
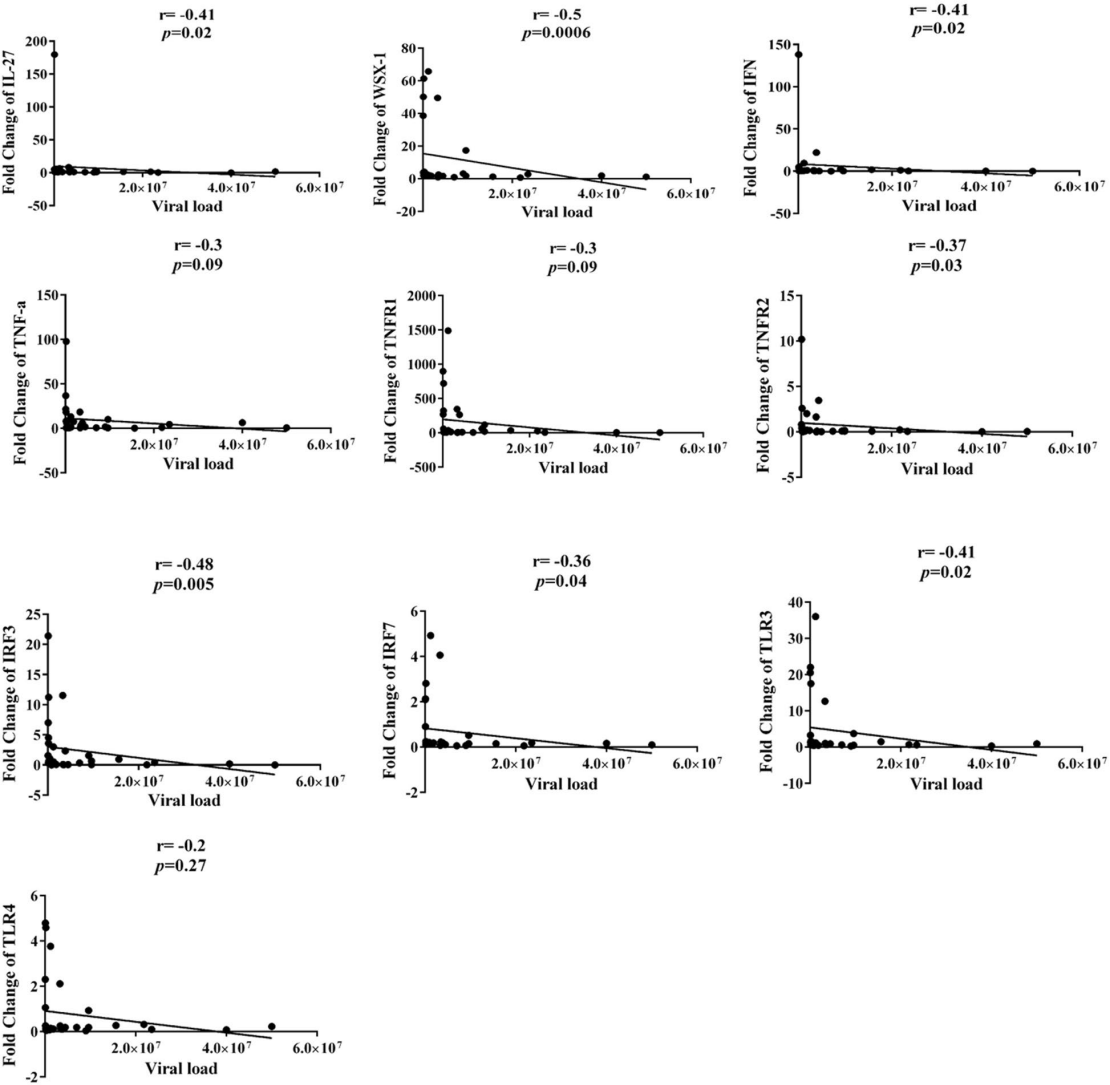
Studying the relation between viral loads and gene expression level in active group, significant negative correlation is detected between most of the studied genes except for TNF-α, TNFR1, and TLR4

The results of studying the relation between viral loads with different gene expression in active group were done using Spearman method. The results of this analysis are summarized in Fig. 4. These analyses revealed that the expression level of most of the studied genes (except for TNF-α, TNFR1, and TLR4) have significant negative correlation with viral load.

## Discussion

During BKVAN, replication of BKV in kidney happens in an uncontrolled manner that cause an increase in the viremia, which might culminate in graft loss. The detailed mechanism of viral persistence and viral reactivation after immunosuppressive conditions remain to be answered [12]. Previously, it was detected that probably due effects of immunosuppression, KTRs with and without BKV viruria had lower amounts of BK-specific T cells compared with healthy patients and BK virus related CD4 and CD8 cells were not detectable in viremic patients [32]. Other researches detected that BK-reactive T cells appears during graft function improvement [16, 33]. These data suggest that the central part of viral infection control might be the restoration of immune competence. However, Chakera et al. could not find any correlation between BK-specific T cells and BK peptides, this indicates that some other issues must be involved in the absence of specific immunity during post-transplantation time [33]. Therefore, by considering that it is not completely clear, how BKV infection can affect the regulation of immune system especially in KT patients, we aimed to study IL-27 as an important pro/anti-inflammatory cytokines and its related pathway genes in this regard.

Cytokines such as IL-27 and other related molecules and receptors in its signaling pathway can have either pro-inflammatory or anti-inflammatory roles in different situations [34]. This molecule by attaching to its receptor (IL-27R) starts a pathway that seems to be necessary for early CD4 T cell differentiation [27]. Furthermore, in activated naive T cells, IL-27 strongly cooperate with IL-12 for effective production of IFN-γ in order to prepare the suitable environment for commitment to a Th1 phenotype [4]. During viral infections, Frank et al. showed that IL-27 induces STAT-1 and -3 in dose-dependent manner for inhibiting not only HCV infection but also HCV/HIV co-infection [31]. Furthermore, in HBV infection IL-27 and IFN-λ1 simultaneously trigger the inhibition of HBV replication [29]. Also, our previous study on the HBV infected liver transplanted patients showed that HBV in the mentioned patients causes over expression of IL-27 gene in comparison to uninfected patients [30].

In its pro-inflammatory pathway, IL-27 can enhance the proliferation of naïve CD4 T cells and production of IFN-γ [35]. Also, in vitro studies in mouse and human CD8 T cells revealed the activation of pSTAT 1–5 and increase in T-bet and IFN-γ production [36]. The effects of IL-27 on B cells and humoral responses are also studied. IL-27 can start class switching in B cells, although in comparison to IFN-γ and IL-4, this phenomenon happens more moderately [37, 38].

Although early studies focused on the capacity of IL-27 to increase TH1 immunity, successive researches using parasitic systems, discovered the IL-27 immune suppressive effects [39]. Studies with different models including viral (influenza) ones [40] show that IL-27 is a critical negative regulator of the pathology associated with these models (such as parasitic, bacterial, viral, autoimmune and others). Subsequently, it is noted that in vivo suppressive effects of IL-27 are related to different anti-inflammatory criteria of IL-27, such as the ability to limit CD4+ cells for IL-2 or IFN-γ production and also, T cell expression of the IL-10 anti-inflammatory cytokine [41–43], which might be related to the ability of NKT cells in producing IL-27 in order to negatively regulate TH2 responses [44]. Also, the facility of IL-27 to attenuate T cell differentiation is not limited to TH1 or TH2 responses, as many reports have revealed that IL-27 modulates TH17 activities, too [42, 45]. Collectively, results imply that, during bacterial or parasitic infections which act like strong polarizing stimuli, the ability of IL-27 to promote TH1-cell responses becomes secondary to its role as a suppressor of effector T-cell proliferation and cytokine production [26].

In our study, the results showed that the mRNA expression level of IL-27 in active group is less than inactive one, significantly. Considering the point that the level of immunosuppressant drugs in the time of study is the same for both viral active or inactive KTRs, the data shows that activation of BKV is strongly controlled by a mechanism that shut downs the expression of IL-27. This causes the immune system to increase the expression of IL-27R in this group of patients probably to compensate the lack of IL-27 through increase in the number of its receptors on the surface of immune cells. The result of this process is observed in the significant increase of IL-27R mRNA expression level in the active group. Although the role of IL-27 in driving CD8+ T cell responses in infectious disease is less studied, initial studies showed that in the absence of IL-27Ra, CD8+ T cell responses are not effective during infection with Trypanosoma cruzi and influenza [40, 46]. Following the BKV activation in patients, the expression of IL-27 decreases by an unknown manner, it seems this molecule cannot start a pro- or anti-inflammatory detectable pathway. In our study, this decrease in expression has been detected in the expression of IFN-γ, whose increase was previously documented in pro-inflammatory pathway of IL-27 activation [4]. Moreover, IL-27 in its anti-inflammatory pathway downregulates the expression of IFN-γ and promotes the expression of the potent anti-inflammatory cytokine IL-10 [41, 42, 47].

Studies specify that only when IL-27 exists from the start in the cultures it can suppress the production of cytokines such as IL-17, IL-4, IL-2 and IFN-γ. Consequently, addition of IL-27 even as early as 24 h after T cell activation results in a decrease in its anti-inflammatory potential. Overviewing the results of these researches suggest that presence of IL-27 only is effective for cytokine production inhibition, while CD4 + Tcells are about to differentiate through receiving activating signals but are still undifferentiated, and it is not about to happen when an active immune response is taking place in vivo [5, 48]. Furthermore, following the stimulation that is produced by BK’s VP1 and LT antigens, the expression of IFN-γ, TNF-α, CD107, and granzyme A and B was the reason of the existence of BK virus specific T cells in the healthy seropositive patients’ blood [23].

The exposition of renal tubular cells to viral products stimulates and activates host receptors such as TLR3 to induce anti-viral and pro-inflammatory responses [49]. Our results emphasize on the production of TLR3 in active patients’ group, which seems to be related to the struggle of the innate immune system for beating the virus, through increasing the production of TLR3 receptors. As TLR4 receptors are engaged in recognition of bacterial and fungal molecules in contrast to intracellular receptors such as TLR3 that recognizes nucleic acids [50], in order to preparing a comparison and elucidating the role of TLR3 in viral infections, we investigated the expression level of TLR4 in our samples. The results showed that the TLR4 expression level in both patient’s groups was less than even control group. These results reinforced the previous results that explained TLR4 does not have any role in viral infections.

Microbial stimuli can induce IL-27 (both EBI3 and IL-27p28 mRNAs) in antigen presenting cells through TLR4 activation [51–53]. Studies showed that in response to TLR4 and TLR3 activation, signals via TRIF (Toll/IL-1R-related domain containing adaptor-inducing IFN) cause the activation of IRF3 and IRF7 that finally results in an increase in the mRNA expression of IL-27 subunits [54, 55]. Also, it is important to know that IRF3 is related to the promoter region of IL-27p28 and not EBI3 [54, 56]. Instead, the promoter region of EBI3 has a consensus sequence for IRF7 attachment [53]. IRF7 is a lymphoid-specific factor and is potentially induced by stimuli such as type I IFNs, TNF-α and lipopolysaccharide in different cell types [57, 58]. Also, the IRF3/IRF7 heterodimer is commonly known to be involved in viral infection, inflammatory diseases [59]. Due to the role of IRF3 and its closely related molecule, IRF7, in IFN production induction the majority of IRF3 and IRF7 studies were devoted to understanding their involvement in cell responses to pathogens, mainly viruses [60]. Our results showed that IRF3 is higher in viral active patients but IRF7 was significantly higher in inactive group, which might be one of the consequences of TNF-α decrease in active patient group. Therefore, production of IRF7 in these patients was not possible.

Finally, ROC curve analysis demonstrated that IL-27 (*p* < 0.001 and AUC = 0.956) and IRF7 (*p* < 0.001 and AUC = 0.982) might be more important candidate for further studies in the field of finding probable biomarkers for filtering BK active infection from inactive ones.

## Conclusion

Analysis showed IL-27 and IRF7 are significantly downregulated in BKV active group in comparison to the inactive one and this means more studies in this field are required in order to elucidate the detailed regulatory function of BK viral infection among kidney transplanted patients. Therefore, these results might be a step forward in finding a pattern which is responsible for reactivation of BK virus in kidney transplanted patients.

## Supplementary Information

**Additional file 1.**

## Data Availability

The datasets used and/or analyzed during the current study are available from the corresponding author on reasonable request.

## Abbreviations

BKV: BK polyomavirus
KT: kidney transplantation
BKVAN: BKV-associated nephropathy
KTR: kidney transplant recipients
IL-27: interleukin-27
EBI3: Epstein-Barr virus induced gene 3
NK: Natural killer
DCs: Dendritic cells
KIR: killer cell immunoglobulin-like receptors
HD5: human defensin 5
IFN-γ: gamma interferon
TNF: tumor necrosis factor
TLR: toll-like receptor
IRF: interferon regulatory transcription factor
TNF-α: tumor necrosis factor- alpha
