# Supplementary material for: The effect of BKV reactivation on cytokines behavior in kidney transplanted patients
Source: BMC Nephrol. 2022 Jan 7;23:20. doi: 10.1186/s12882-021-02645-y (PMC8739991; doi:10.1186/s12882-021-02645-y)
Supplement: Supplementary file 1 — Additional file 1. [file 12882_2021_2645_MOESM1_ESM.docx]

**Supplementary Information**

**Title: The effect** **of BKV reactivation on cytokines behavior in kidney transplanted patients**

**Authors:** Zahra Rahimi, Ramin Yaghobi, Afsoon Afshari, Jamshid Roozbeh, Mohammad Javad Mokhtari, Ali Malek Hosseini

**Table S1-** The viral loads details of KTRs with active BKPyV infection

| **Patient No.** | **Ct value** | **DNA viral load***** (copy/ml)** | **Patient No.** | **Ct value** | **DNA viral load (copy/ml)** |
| --- | --- | --- | --- | --- | --- |
| **1** | 27 | 8x10^5^ | **17** | 32 | 8.5 x10^5^ |
| **2** | 28 | 2.91x10^5^ | **18** | 27 | 2.78 x10^5^ |
| **3** | 27 | 18.55 x10^5^ | **19** | 24 | 80 x10^5^ |
| **4** | 24 | 38.4 x10^5^ | **20** | 27 | 4.045 x10^5^ |
| **5** | 20 | 400 x10^5^ | **21** | 25 | 1.335 x10^5^ |
| **6** | 26 | 10 x10^5^ | **22** | 26 | 12.5 x10^5^ |
| **7** | 31 | 1.345 x10^5^ | **23** | 23 | 32.8 x10^5^ |
| **8** | 21.5 | 235 x10^5^ | **24** | 20 | 96 x10^5^ |
| **9** | 23 | 90 x10^5^ | **25** | 21.5 | 1.43 x10^5^ |
| **10** | 21 | 44.5 x10^5^ | **26** | 34 | 4.8 x10^5^ |
| **11** | 20 | 218 x10^5^ | **27** | 26 | 8.8 x10^5^ |
| **12** | 22 | 33.5 x10^5^ | **28** | 26 | 12 x10^5^ |
| **13** | 17 | 500 x10^5^ | **29** | 25 | 1.95 x10^5^ |
| **14** | 28 | 1.76 x10^5^ | **30** | 20 | 96 x10^5^ |
| **15** | 24 | 35 x10^5^ | **31** | 27 | 6.9 x10^5^ |
| **16** | 22 | 156.5 x10^5^ |  |  |  |

*****The viral load mean of studied patients is 69.8 x10^5^
